# A BAX/BAK and Cyclophilin D-Independent Intrinsic Apoptosis Pathway

**DOI:** 10.1371/journal.pone.0037782

**Published:** 2012-06-12

**Authors:** Sebastián Zamorano, Diego Rojas-Rivera, Fernanda Lisbona, Valentina Parra, Felipe A. Court, Rosario Villegas, Emily H. Cheng, Stanley J. Korsmeyer, Sergio Lavandero, Claudio Hetz

**Affiliations:** 1 Biomedical Neuroscience Institute, Faculty of Medicine, University of Chile, Santiago, Chile; 2 Center for Molecular Studies of the Cell, Institute of Biomedical Sciences, University of Chile, Santiago, Chile; 3 Faculty of Chemical & Pharmaceutical Sciences, University of Chile, Santiago, Chile; 4 Millennium Nucleus for Regenerative Biology, Faculty of Biology, P. Catholic University of Chile, Santiago, Chile; 5 Human Oncology and Pathogenesis Program, Memorial Sloan-Kettering Cancer Center, New York, New York, United States of America; 6 Dana–Farber Cancer Institute, and Harvard Medical School, Boston, Massachusetts, United States of America; 7 Cardiology Division, Department of Internal medicine, University of Texas Southwestern Medical Center, Dallas, Texas, United States of America; 8 Harvard School of Public Health, Boston, Massachusetts, United States of America; 9 Neurounion Biomedical Foundation, Santiago, Chile; Roswell Park Cancer Institute, United States of America

## Abstract

Most intrinsic death signals converge into the activation of pro-apoptotic BCL-2 family members BAX and BAK at the mitochondria, resulting in the release of cytochrome c and apoptosome activation. Chronic endoplasmic reticulum (ER) stress leads to apoptosis through the upregulation of a subset of pro-apoptotic BH3-only proteins, activating BAX and BAK at the mitochondria. Here we provide evidence indicating that the full resistance of BAX and BAK double deficient (DKO) cells to ER stress is reverted by stimulation in combination with mild serum withdrawal. Cell death under these conditions was characterized by the appearance of classical apoptosis markers, caspase-9 activation, release of cytochrome c, and was inhibited by knocking down caspase-9, but insensitive to BCL-X_L_ overexpression. Similarly, the resistance of BIM and PUMA double deficient cells to ER stress was reverted by mild serum withdrawal. Surprisingly, BAX/BAK-independent cell death did not require Cyclophilin D (CypD) expression, an important regulator of the mitochondrial permeability transition pore. Our results suggest the existence of an alternative intrinsic apoptosis pathway emerging from a cross talk between the ER and the mitochondria.

## Introduction

Apoptosis is a conserved cell death mechanism essential for normal development and tissue homeostasis in multicellular organisms. Although apoptosis presumably participates in the development of most cell lineages, alterations in the expression of apoptosis-regulatory proteins is implicated in the initiation of a variety of human diseases, including autoimmunity, immunodeficiency, cancer, and neurodegenerative diseases, among others [1], [2]. The BCL-2 family of proteins is a group of upstream regulators of the caspase cascade, comprised of both pro- and anti-apoptotic components [1], [2]. BCL-2 family members are defined by the presence of up to four α-helical conserved **B**CL-2 **h**omology (BH) domains. Pro-apoptotic BCL-2 family members can be further subdivided into more highly conserved, “multidomain” members displaying homology in the BH1, BH2 and BH3 domains (i.e. BAX and BAK), and the “BH3-only” members which contain a single BH domain critical for activation of apoptosis.

Genetic and biochemical studies indicate that BAX and BAK function in concert as a major core of the intrinsic apoptosis pathway at the mitochondria [3], [4]. Upstream BH3-only proteins respond to particular apoptotic signals and subsequently trigger the conformational activation of BAX and BAK, inducing their intramembranous homo-oligomerization and resultant mitochondrial outer membrane permeabilization (MOMP) [5]. MOMP is a key step for the release of cytochrome c and the assembling of the apoptosome [5], [6]. Besides, the BH3-only proteins can be functionally separated into two subtypes: (i) activators (i.e. tBID, BIM, and PUMA) that directly engage BAX and BAK to trigger cytochrome c release, but are sequestered by anti-apoptotic BCL-2 molecules; and (ii) sensitizers or inactivators (i.e. BAD and NOXA) that only bind to and antagonize anti-apoptotic BCL-2 members to release activator BH3-only proteins (examples in [7]–[11]). Alternatively, differential binding to anti-apoptotic proteins may explain the action of activator and sensitizer/inactivator BH3-only proteins [12] or combination of both models [11], [13], [14].

Under certain conditions, cytochrome c release occurs independent of BAX and BAK through opening of the mitochondrial permeability transition pore (PTP), a non-specific pore in the inner mitochondrial membrane (see reviews in [15]–[17]). Opening of the PTP is observed under conditions of mitochondrial calcium overload, especially when accompanied by oxidative stress, elevated phosphate concentrations and adenine nucleotide depletion, enabling free passage into the mitochondria of molecules of <1.5 kDa [15]–[17]. Opening of the PTP leads to dissipation of the mitochondrial transmembrane potential (ΔΨ_m_) and an influx of solutes. This causes expansion of the matrix, resulting in sufficient swelling to rupture the outer mitochondrial membrane and cytochrome *c* release. However, dissipation of ΔΨ_m_ can also lead to a sudden decrease in ATP levels, triggering necrotic cell death. Although the molecular identity of PTP remains uncertain, different components are proposed including voltage-dependent anion channel (VDAC), the adenine nucleotide translocator, the mitochondrial phosphate carrier, and Cyclophilin D (CypD), a cyclosporin A target [15]–[17]. Studies using knockout cells for putative components of the PTP confirmed only a functional role for CypD in PTP-mediated cell death *in vitro* and *in vivo* as we and other described [18]–[21]. Remarkably, physical interactions between BCL-2 family members and components of the PTP are also reported, suggesting that BCL-2-related proteins may facilitate PTP under certain conditions, possibly forming mixed protein complexes with membrane permeabilizing activities (reviewed in [17]).

Interestingly, Y. Tsujimoto’s group reported the engagement of apoptosome dependent apoptosis that was independent of the expression of BAX, BAK and CypD, a phenomena initiated when death stimulation was induced by the simultaneous exposure of cells arachidonic acid and the ionophore A23187 [22], stimuli known to alter endoplasmic reticulum (ER) calcium homeostasis among other effects. Cell death after arachidonic acid and A23187 treatment was dependent on the activity of an unknown serine protease. In addition to operate as a major calcium reservoir of the cell, the ER serves as a specialized compartment for protein folding along the secretory pathway. A number of conditions interfere with protein folding at the ER lumen leading to the accumulation of unfolded or misfolded proteins, a cellular condition referred to as “ER stress” [23]. Cells adapt to ER stress through the engagement of the Unfolded Protein Response (UPR), an integrated signaling pathway that controls protein folding at the ER [24], [25].

Under chronic ER stress conditions, cells undergo apoptosis mediated by the activation of BAX and BAK at the mitochondria [26]. BAX and BAK double deficient (DKO) cells [3], [27], [28] and conditional DKO mice [29] are highly resistant to death stimuli dependent on ER injuries. ER stress-induced apoptosis is initiated by the activation of several BH3-only protein including BIM, PUMA and NOXA, and is antagonized by the expression of BCL-2 or BCL-X_L_ [10], [30], [31]. In this article, we provide evidence indicating the occurrence of cytochrome c release and caspase-9-dependent apoptosis in BAX and BAK DKO cells when ER stress is stimulated in combination with mild serum withdrawal. Interestingly, BAX and BAK-independent cell death did not require CypD expression, did not involve mitochondrial swelling, and was insensitive to serine protease inhibitors. Our results are indicative of the existence of an alternative intrinsic cell death pathway that is independent of BAX/BAK and CypD expression that possibly triggers apoptosome-mediated cell death.

## Results

### Mild Serum Withdrawal Recovers the Susceptibility of BAX and BAK DKO Cells to ER Stress-induced Cell Death, but Not to other Intrinsic Death Stimuli

**Figure 1:**
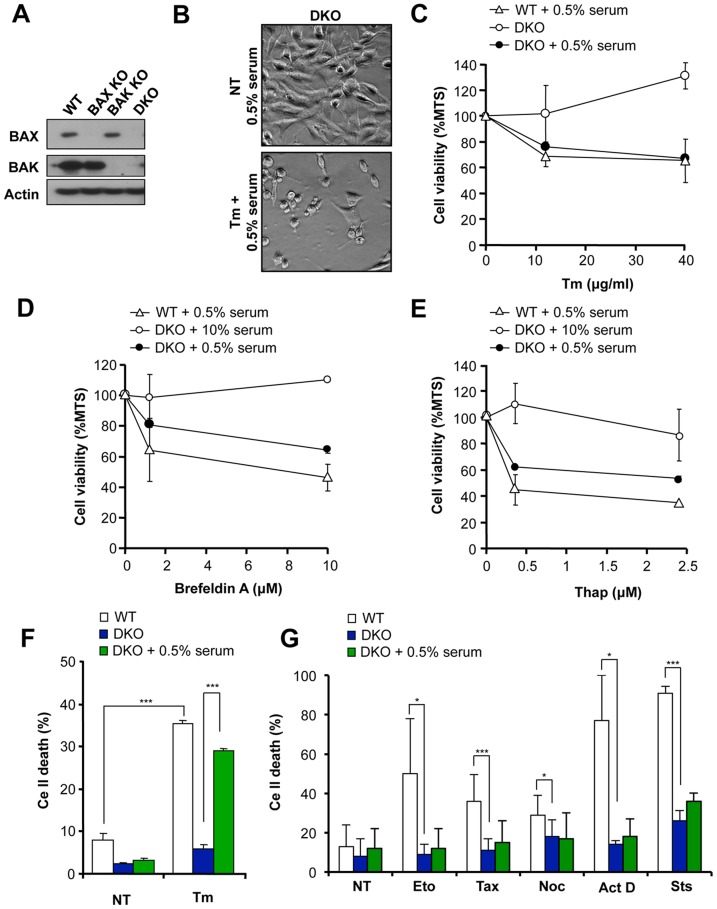
Serum withdrawal recovers the susceptibility of BAX and BAK DKO cells to ER stress-induced cell death. (**A**) Expression levels of BAX and BAK were determined in murine embryonic fibroblast from wild type (WT), BAX or BAK single knockout (KO) or BAX and BAK DKO (DKO) cells by Western blot. As a loading control actin levels was determined. (**B**) Phase contrast images of BAX/BAK DKO cells treated or not (NT) with 10 µg/ml Tm for 16 h in cells pre-exposed for 2 h to cell culture media containing 0.5% serum. (**C**) BAX and BAK DKO MEFs were treated with indicated concentrations of Tm, (**D**) Brefeldin A or (**E**) Thap in cells grown in regular cell culture media or pre-treated in media containing 0.5% serum for 2 h. WT cells grown in media containing 0.5% serum were monitored as control (see additional controls in Figure S1). After 24 h of treatment, cell viability was analyzed with the MTS assay. Mean and standard deviations are presented of triplicates of a representative experiment. (**F**) BAX and BAK DKO cells were pre-incubated for 2 h in cell culture media containing 0.5% serum or grown with regular cell culture media (10% serum), and then treated with a panel of different intrinsic death stimuli, including 10 µg/ml Tm. Untreated cells (NT) were used as control. After 24 h of treatment cell death was monitored by propidium iodide (PI) staining and FACS analysis. Results represent average and standard deviation of six independent experiments. Student’s T-test was used to analyze statistical significance between cells treated with Tm in the presence of 10% or 0.5% serum. (***: *p*<0.001). (**G**) Alternatively, cells were analyzed as described in F and treated with 20 µM etoposide (Eto), 1 µM taxol (Tax), 10 µM nocodazole (Noc), 10 µg/ml actinomycin D (Act D) or 0.1 µM staurosporine (Sts). Results represent average and standard deviation of 4 independent experiments. Student’s T-test was used to analyze statistical significance between cells treated with these drugs in the presence of 10% or 5% of serum (*: *p*<0.05; ***: *p*<0.001).

We previously reported an impaired ability of BAX and BAK DKO murine embryonic fibroblasts (MEFs) to activate the ER stress sensor IRE1α, associated with diminished downstream cJun-N terminal kinase (JNK) signaling [29]. To assess the impact of BAX and BAK deficiency on JNK phosphorylation in that study, we stimulated cells in the presence of low serum concentrations (0.5–2% serum) for 2 h to decrease basal phosphorylation levels, and then exposed cells with the ER stress agent tunicamycin (Tm, inhibitor of N-linked glycosylation). Interestingly, when cells were cultured under these conditions for prolonged time (>16 h), we noticed morphological changes reminiscent of apoptosis in BAX and BAK DKO cells, including the appearance of blebs at the cell surface, cell shrinkage and detachment from the cell culture plate (Figure 1A and 1B. C. Hetz and S.J. Korsmeyer, 2004, unpublished observations).

To further characterize the death mechanism of BAX and BAK DKO cells under combined stimulation of ER stress and mild serum withdrawal, cells were treated with a panel of drugs that perturb ER homeostasis including Tm, brefeldin A (an inhibitor of ER to Golgi trafficking), or thapsigargin (Thap, inhibitor of the ER calcium ATPase SERCA) in the presence of regular cell culture media containing 10% serum or pre-treated for 2 h with media containing 0.5% serum. After 24 h, relative cell number was monitored with the MTS assay. To our surprise, resistance of BAX and BAK DKO cells to ER stress was reverted when stimulation was performed under conditions of mild serum withdrawal (Figure 1C-E and Figure S1). Serum withdrawal alone did not induce cell death of BAX and BAK DKO cells, even after three days of treatment (not shown) as previously described [3]. Similar results were observed when cell death was monitored using propidium iodide (PI) staining and FACS analysis (Figure 1F).

In order to define whether mild serum withdrawal sensitizes DKO cells to other intrinsic death stimuli, cells were treated with a variety of pro-apoptotic agents that are non-ER stress related. These include etoposide (DNA damage agent), taxol and nocodazole (both alter microtubule dynamics), and actinomycin D (an inhibitor of protein synthesis). Unexpectedly, the resistance of BAX/BAK DKO cells to each of these pro-apoptotic agents was not reverted by mild serum withdrawal (Figure 1G). Similarly, serum withdrawal did not enhance the susceptibility of BAX/BAK DKO cells to engagement of death receptors since a similar rate of cell death was observed after treatment with TNFα when compared to control cells grown in the presence of 10% serum (∼80% cell death, not shown). These results suggest that combinatorial stimulation between ER stress and mild serum withdrawal engages a BAX/BAK-independent cell death pathway.

### Caspase-dependent Apoptosis in BAX and BAK DKO Cells Undergoing ER Stress in Conjunction with Serum Withdrawal

**Figure 2:**
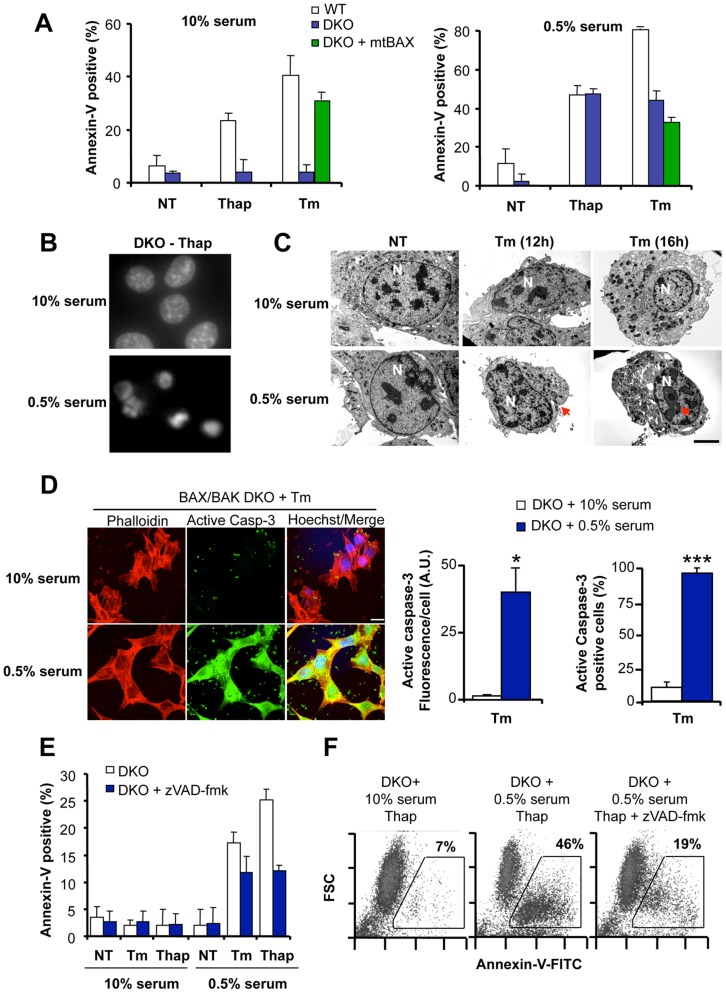
BAX and BAK double deficient cells undergo caspase-dependent apoptosis under conditions of ER stress in conjunction with serum withdrawal. (**A**) Left panel: WT, BAX/BAK DKO cells or DKO cells stably reconstituted with mitochondrial-targeted BAX (mtBAX) were treated with 10 µM Thap or 10 µg/ml Tm under normal growing conditions (10% serum). After 12 h, apoptosis was monitored by Annexin-V-FITC and PI staining followed by FACS analysis. Mean and standard deviations are presented of three determinations. Right panel: Alternatively, experiments were performed in cells pre-treated for 2 h with cell culture media containing 0.5% serum, followed by the addition of ER stress agents. (**B**) BAX/BAK DKO cells were exposed to 10 µM Thap in the presence of cell culture media containing 10% serum or pretreated in media containing 0.5% serum, and then cells were stained with Hoechst to visualize nuclear morphology by fluorescent microscopy. (**C**) Cell morphology was monitored in BAX/BAK DKO cells treated with Tm for 12 h or 16 h under normal growing conditions or in cells pretreated for 2 h with media containing 0.5% serum. Untreated (NT) cells are also presented as control. Red arrowheads indicate classical apoptosis morphological features including chromatin condensation and fragmentations. In addition cell shrinkage is observed in the analysis. N: nucleus. (**D**) BAX/BAK DKO cells were exposed to 10 µg/ml Tm in the presence of cell culture media containing 10% serum (top panel) or pretreated in media containing 0.5% serum (Botton panel). After 10 h, active caspase 3 (green) was evaluated by indirect immunofluorescence. To visualize cells, actin was monitored with Phalloidin-Rhodamine staining, and nuclei were stained with Hoechst (blue). Images were acquired with a confocal microscope and represent the result of four independent experiments. Left panel: Quantification of the levels of active caspase-3 per cell by measuring fluorescent pixel intensity/area (A.U.) or the percentage of cells with positive active caspase-3 signal over a cutoff definer in non treated cells. Mean and standard deviation is presented of four experiments. Student’s T-test was used to analyze statistical significance between both groups (*: *p*<0.05; ***: *p*<0.001). (**E**) BAX/BAK DKO cells were pretreated for 30 min with 10 µM zVAD-fmk or left untreated in media containing 10% or 0.5% serum (pre-incubation of 2 h), and then exposed to 10 µg/ml Tm or 10 µM Thap. After 12 h, Annexin-V-FITC staining was evaluated by FACS analysis. Mean and standard deviation is presented of three determinations. (**F**) In parallel, the correlation between Annexin-V-FITC staining and cell shrinkage was evaluated by FACS by monitoring forward scatter parameter in cells described in (**E**). Data represent the results of three independent experiments.

To determine the mechanism involved in the death of BAX/BAK DKO cells undergoing ER stress under mild serum withdrawal conditions, we performed Annexin-V-FITC and PI staining. As previously described [3], [27], [28], DKO cells grown in cell culture media containing 10% serum were completely resistant to apoptosis induced by ER stress (Figure 2A). Reconstitution of DKO cells with mitochondria-located BAX restored the sensitivity of these cells to Tm-mediated cell death (Figure 2A, left panel), confirming the requirement of BAX expression at the mitochondria to initiate apoptosis by ER-related injuries [27]. BAX and BAK DKO cells exposed to Tm or Thap in conjunction with mild serum withdrawal underwent apoptosis measured after Annexin-V-FITC staining (Figure 2A, right panel).

We confirmed the appearance of apoptosis markers in BAX/BAK DKO cells treated with Tm under mild serum withdrawal by the visualization of chromatin condensation and fragmentation by nuclear staining with Hoechst (Figure 2B). We also detected ultrastructural features of apoptosis under our experimental conditions using electron microscopy (EM), associated with the appearance of condensed and fragmented nuclei, in addition to cell shrinkage (Figure 2C). Consistent with these results, a strong activation of caspase-3 was detected using immunofluorescence when DKO cells were co-stimulated with Tm and low serum concentrations (Figure 2D). Minimal activation of caspase-3 was detected in DKO cells treated with Tm under normal growing conditions (Figure 2D).

In order to test the possible involvement of caspases in the death of BAX and BAK DKO cells, we pre-incubated cells with the pan-caspase inhibitor zVAD-fmk for 30 minutes before treatment with Tm or Thap in the presence of 0.5% serum in the cell culture media. zVAD-fmk treatment drastically reduced the levels of Annexin-V-FITC staining and cell shrinkage observed in DKO cells (Figure 2E). Cell shrinkage was also observed by FACS analysis of DKO cells reflected on a decrease in the forward side scattering (FSC) parameter, which directly correlated with the levels of phosphatidylserine exposure at the cell surface (Figure 2F). Thus, mild serum withdrawal fully reverts the resistance of BAX and BAK DKO cells to ER stress-mediated apoptosis.

### Cell Death in BAX and BAK DKO Cells is Associated with Cytochrome c Release, a Drop in ΔΨ_m_, and is Mediated by Caspase-9

**Figure 3:**
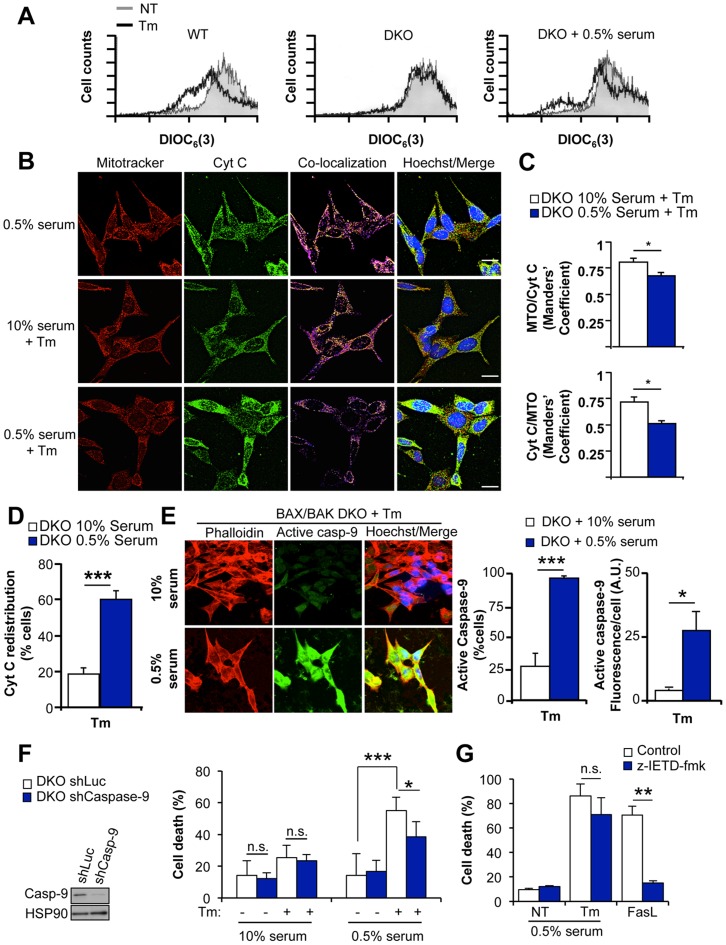
ER stress triggers cytochrome c redistribution and caspase-9-dependent cell death. (**A**) WT and BAX/BAK DKO cells were treated with 10 µg/ml Tm or left untreated (NT) in regular cell culture media or in cells pre-treated with media containing 0.5% serum for 2 h. After 8 h of treatment, ΔΨ_m_ was determined by DIOC_8_(3) staining and FACS analysis. (**B**) BAX/BAK DKO cells were exposed to 10 µg/ml Tm in the presence of cell culture media containing 10% serum (top panel) or pre-treated with media containing 0.5% serum (Botton panel). After 10 h, cells were stained with Mitotracker orange (red), fixed and cytochrome C (Cyt C, green) distribution visualized by indirect immuofluorescence. DNA was stained with Hoechst (blue). Images were acquired by confocal microscopy and are representative of four independent experiments. Scale bar is 25 µm. (**C**) Quantification of the Mandeŕs colocalization coefficient MTO/CytC (fraction of mitrotracker orange signal in co-localizing with cytochrome C signal) or CytC/MTO (fraction of the cytochrome C co-localizing mitotracker orange). Student’s T-test was used to analyze statistical significance (*: *p*<0.05). (**D**) Quantification of the percentage of cells with redistributed cytochrome C (CytC) experiments described in C (***: *p*<0.001). (**E**) Left panel: BAX/BAK DKO cells exposed to 10 µg/ml Tm for 10 h in the presence of cell culture media containing 10% (top panel) or 0.5% (bottom panel) serum were fixed and active caspase 9 (green) was evaluated by indirect immunofluorescence. Actin was monitored with Phalloidin-Rhodamine (red) and nuclei were stained with Hoechst (blue). Images are representative of four independent experiments. Scale bar is 25 µm. Right panel: Quantification of active caspase 3 fluorescence per cell (Arbitrary units: A.U.) and the percentage of active-caspase 3-positive cells. Data represents mean and standard deviation of four independent experiments performed in duplicates. Student’s T-test was used to calculate statistical significance (*: *p*<0.05; ***: *p*<0.001). (**F**) BAX and BAK DKO cells were stably transduced with a lentiviral vector expressing an shRNA against *pro-caspase-9* mRNA (shCaspase-9) or a control shRNA against the luciferase mRNA (shLuc), and pro-caspase-9 protein levels were determined by Western blot. HSP90 levels were monitored as loading control. Then, the cells were treated with 10 µg/ml Tm in cells grown under normal cell culture media or pre-treated for 2 h with media containing 0.5% serum. Cell death was determined after 24 h by PI staining and FACS analysis. Data represents mean and standard deviation of three independent experiments performed in duplicates. Student’s T-test was used to analyze statistical significance between shLuc and shCaspase-9 cells treated with Tm (*: *p*<0.05; n.s.: no significant). (**G**) BAX and BAK DKO cells were pre-incubated in media containing 0.5% serum for 2 h. Then, the cells were exposed to 10 µg/ml Tm for 24 h in present or absence of 25 µM z-IETD-fmk (caspase-8 inhibitor). As positive control, DKO cells were exposed to 1 µg/ml Fas ligand (FasL) as positive control of working Z-IETD-FMK. The cell death was monitored by propidium iodide (PI) staining and FACS analysis. Results represent average and standard deviation of three independent experiments. Student’s T-test was used to analyze statistical significance (**: *p*<0.01; n.s.: no significant).

To define the potential involvement of mitochondria in the occurrence of BAX/BAK-independent apoptosis, we first measured ΔΨ_m_ in cells undergoing ER stress. As expected, whereas WT MEFs presented a clear drop on ΔΨ_m_ after Tm treatment, BAX/BAK DKO cells showed no decrease on ΔΨ_m_ as determined by DIOC_8_(3) staining and FACS analysis (Figure 3A). This phenotype was partially reverted by performing treatments with Tm in conjunction with serum withdrawal (Figure 3A). To further examine the contribution of mitochondria in the cell death process, we visualized the distribution of cytochrome c in BAX/BAK DKO cells by immunofluorescence. As shown in Figure 3B, cytochrome c redistribution was observed in DKO cells treated with Tm in conjunction with serum withdrawal, resulting in the partial loss of the punctuate pattern characteristic of mitochondria-located cytochrome c into a more diffuse cytoplasmic pattern (Figure 3B). Quantification of four independent experiments revealed a significant reduction in the Manders co-localization index between cytochrome c and a mitochondrial tracker when ER stress was stimulated in conjunction with mild serum withdrawal (Figure 3C). Moreover, at the population level, around 60% of BAX/BAK DKO cells presented cytochrome c redistribution using this imaging analysis after ER stress and serum withdrawal stimulation (Figure 3D).

We then monitored the possible assembly of the apoptosome by monitoring the activation of caspase-9 using immunofluorescence using a specific antibody against active caspase-9. A consistent activation of caspase-9 was detected in most BAX and BAK DKO cells when treated with Tm and low serum concentration in terms of signal intensity or percentage of positive cells (Figure 3E). To define the functional contribution of the canonical mitochondrial-apoptotic pathway in the death of BAX/BAK DKO cells, we knocked down the expression of pro-caspase-9 using stable delivery of a short hairpin RNA (shRNA) with lentiviral vectors, which only partially reduced pro-caspase-9 levels (Figure 3F). As control, an shRNA construct against *luciferase* mRNA was employed. Knocking down pro-caspase-9 expression significantly decreased the rate of cell death observed in BAX/BAK DKO cells treated with Tm in conjunction with serum withdrawal (Figure 3F, right panel). As additional control, we also tested the possible contribution of caspase-8 to the death process of BAX/BAK DKO cells. Cells were pre-incubated with 40 µM of the caspase-8 inhibitor z-IETD-fmk for 30 min, and the levels of cell death were evaluated by FACS. z-IETD-fmk did not significantly reduce ER stress-induced apoptosis under conditions of mild serum withdrawal (Figure 3G). In contrast, z-IETD-fmk efficiently blocked FasL-induced apoptosis in BAX/BAK DKO cells (Figure 3G). Together, these results suggest an active role of the apoptosome in the death of BAX and BAK DKO cells after stimulation of ER stress in the presence of mild serum withdrawal.

### Cell Death in BAX and BAK DKO Cells is Insensitive to BCL-X_L_ Overexpression

**Figure 4:**
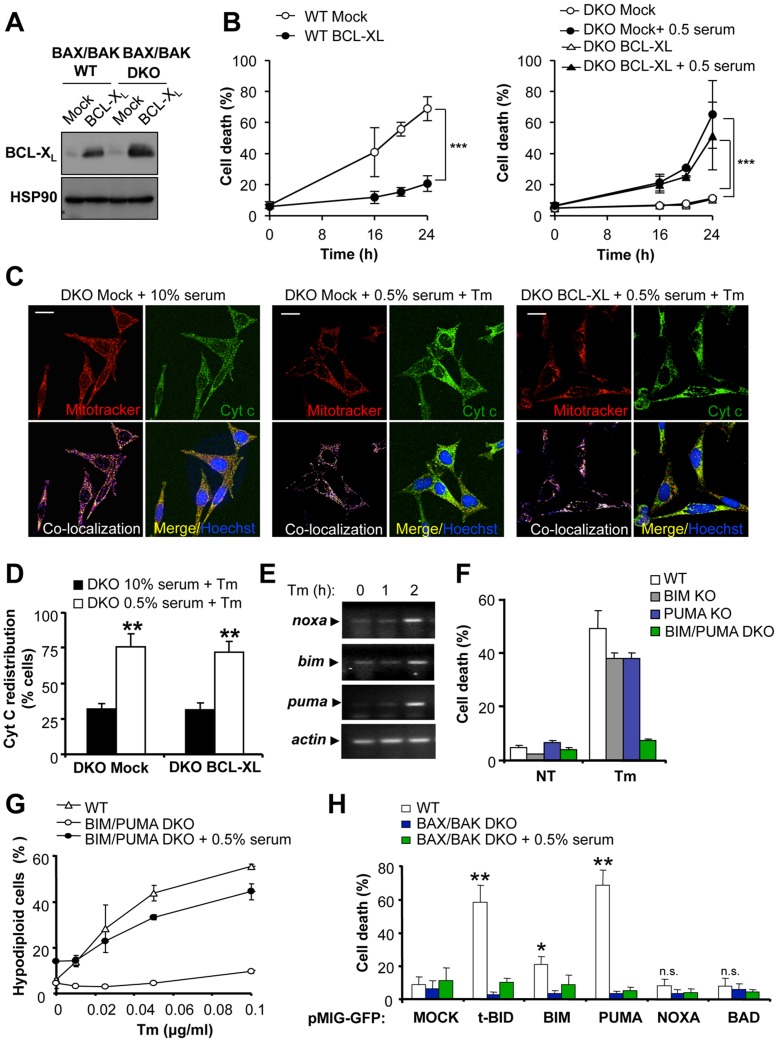
BAX and BAK-independent cells death induced by ER stress and serum withdrawal is not affected by BCL-X_L_ overexpression or by BH3-only proteins. (**A**) WT and BAX/BAK DKO MEFs were stably transduced with retroviruses expressing human BCL-X_L_ or empty vector (Mock) and the expression levels of BCL-X_L_ was evaluated by Western blot analysis. As loading control, HSP90 levels were determined. (**B**) Then the susceptibility of cells presented in (A) to cell death was assessed after treatments with 10 µg/ml Tm in cells pretreated in cell culture media containing 0.5% serum for 2 h or grown with regular cell culture media. Cell death was monitored by PI staining and FACS analysis at 16, 20 and 24 h. Mean and standard deviation is presented of three independent experiments. Two-way ANOVA was used to analyze statistical significance between MOCK and BCL-XL cells (***: *p*<0.001). (**C**) Cells described in A were treated with 20 µg/ml Tm in the presence of cell culture media containing 10% serum (top panel) or pre-treated with media containing 0.5% serum (Botton panel). After 10 h, cells were stained with Mitotracker orange (red), fixed and cytochrome C distribution visualized by indirect immunofluorescence (Cyt C, green). DNA was stained with Hoechst (blue). Images were acquired by confocal microscopy and are representative of four independent experiments. Scale bar is 25 µm. (**D**) Quantification of the percentage of cells with redistributed cytochrome C (CytC) experiments described in C (**: *p*<0.01). (**E**) The mRNA levels of *bim*, *puma* and *noxa* were analyzed by semi-quantitative RT-PCR in total cDNA from MEFS treated with 10 µg/ml Tm for indicated time points. *Beta-actin* levels were determined as loading control. (**E**) WT, BIM KO, PUMA KO, and BIM and PUMA double deficient (DKO) cells were treated with 0.5 µg/ml Tm for 24 h, and then cell death was evaluated after PI staining and FACS analysis. Data represents two independent experiments performed in triplicates. Mean and standard deviation is presented of a representative experiment. (**F**) BIM and PUMA DKO cells and parental WT controls cells were treated with indicated concentrations of Tm as described in (**E**). After 24 h, apoptosis was determined by quantifying cells with hypo-diploid DNA content after PI staining of permeabilized cells and FACS analysis. Mean and standard deviation is presented. (**G**) WT and BAX/BAK DKO cells were transduced with pMIG-GFP retroviruses expressing tBID-HA, BIM-HA, PUMA-HA, NOXA-HA, BAD-HA, or empty vector (MOCK). After 8 h of viral transduction, serum in the culture media was decreased to 0.5% or replaced by media containing 10% serum. Then, cell viability was analyzed after 24 h by PI staining and FACS analysis. Transduction efficiency in each experiment was monitored by detecting GFP expression by FACS in the same experiments (example in Figure S2). Data is the average and standard deviation of three independent experiments. *p* value to compare the rate of cell death in WT cells transduced with BH3-only protein-expressing viruses versus a MOCK transduced WT cells were calculated by Student’s t-test (*: *p*<0.05; **: *p*<0.001; n.s.: non significant). Comparison between DKO cells transduced with retroviruses in the presence or absence of serum were in all cases non significant.

Anti-apoptotic BCL-2 family members are potent inhibitors of intrinsic cell death by antagonizing BH3-only proteins. We stably transduced BAX and BAK WT and DKO cells with retroviruses expressing BCL-X_L_ or the empty vector (Figure 4A) and then assessed their susceptibility to cell death after ER stress/mild serum withdrawal stimulation. As expected, BCL-X_L_ overexpression dramatically protected WT cells against ER stress-mediated apoptosis (Figure 4B, left panel). However, no reduction in the amount of cell death was observed in BAX/BAK DKO cells overexpressing BCL-X_L_ after 24 h of treatment with Tm in conjunction with serum withdrawal (Figure 4B). Similarly, time course analysis revealed a similar kinetic of cell death in BAX and BAK DKO control and BCL-X_L_ overexpressing cells (Figure 4B). In agreement with these results, BCL- X_L_ overexpression did not alter the release of cytochrome c in BAX/BAK DKO cells exposed to similar conditions (Figure 4C), which was confirmed after quantification and statistical analysis (Figures 4D and S2A).

In addition to BAX and BAK, the pro-apoptotic multidomain protein, BCL-2 Ovarian Killer (BOK), remains poorly characterized [32]. We monitored the levels of *bok* mRNA in WT and BAX/BAK DKO MEFs by quantitative RT-PCR. A very low abundance of *bok* mRNA was observed at basal levels in MEFs, which was not altered under conditions of ER stress and serum withdrawal (Figure S3). As positive control we monitored ovarian tissue (Figure S3) [33], [34].

### The Resistance of BIM and PUMA Double Deficient Cells to ER Stress-mediated Apoptosis is Reverted by Mild Serum Withdrawal

BIM, PUMA and NOXA, are strongly induced at the transcriptional level in cells undergoing prolonged ER stress, and cells deficient for these genes are partially resistant to ER stress-related injuries [30], [35], whereas *bim* and *puma* double deficiency drastically protects against ER stress [11]. Based on these findings, we studied the potential role of BIM and PUMA on ER stress-mediated cell death under conditions of mild serum withdrawal. After confirming the upregulation of BIM, PUMA, and NOXA mRNA levels in MEFs undergoing ER stress (Figure 4E), we then investigated the functional role of BIM and PUMA on cell death using a gene ablation approach. BIM or PUMA single knockout cells were slightly resistant to Tm treatment when compared with WT control cells (Figure 4F). In sharp contrast, BIM and PUMA DKO cells were highly resistant to mild concentrations of Tm (Figure 4F), similar to the phenotype described in BAX/BAK DKO cells. Interestingly, the protective effects of *bim* and *puma* ablation were fully reverted when cell death stimulation was performed together with mild serum withdrawal (Figure 4G).

It has be been suggested that the BH3-only protein BAD has pro-apoptotic activities independent of BAX/BAK [36]. To complement our experiments, we determined the possible pro-apoptotic effects of BH3-only proteins after enforced expression in BAX/BAK DKO MEFs under conditions of serum withdrawal. Using the pMIG-GFP bicistronic retroviral system, we transiently expressed a full panel of different BH3-only proteins including tBID, BIM, PUMA, NOXA, and BAD (Figure 4H). As previously reported [7], [37], the overexpression of the activator BH3-only proteins tBID, BIM and PUMA triggers cell death in WT MEFs, which was dependent on the expression of BAX and BAK, whereas overexpression of NOXA and BAD did not affect cell viability (Figures 4H, S2B and S2C). No cell death was observed after the expression of these BH3-only proteins in BAX/BAK DKO cells when serum in the cell culture was decreased to 0.5% (Figure 4G). We controlled the efficiency of retroviral transduction in all the experiments by monitoring GFP expression by FACS analysis, which was in the range of ∼40–60% efficiency (Figure S3B). Taken together, these results suggest that BH3-only proteins are not involved in the occurrence of BAX/BAK-independent apoptosis in our experimental system.

### Assessment of the Contribution of UPR Signaling in the Death of BAX and BAK DKO Cells

**Figure 5:**
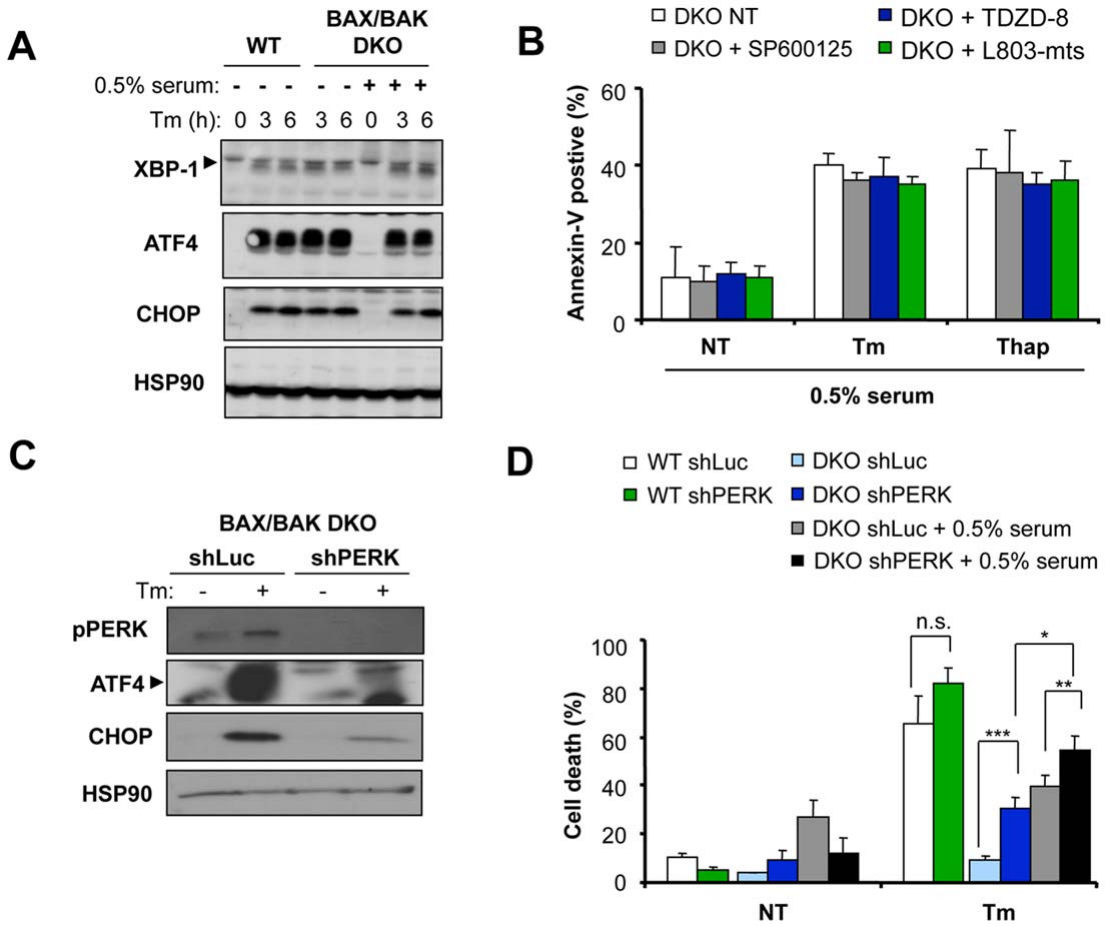
UPR signaling in BAX/BAK DKO cells undergoing ER stress and mild serum withdrawal conditions. (**A**) WT and BAX/BAK DKO cells were treated with 10 µg/ml Tm under regular growing conditions or in cells pre-incubated for 2 h with media containing 0.5% serum. After indicated time points, the expression levels of the UPR transcription factors XBP-1, ATF4 and CHOP were assessed by Western blot analysis. As control, the levels of HSP90 were also determined. (**B**) BAX/BAK DKO cells were pre-treated with 1 µM of the JNK inhibitor SP600125 or 10 µM two different GSK3-β inhibitors (TDZD-8 and L803-mts) for 30 min and then treated with 10 µg/ml Tm or 10 µM Thap under regular growing conditions or in cells pre-treated for 2 h with media containing 0.5% serum. After 16 h, Annexin-V-FITC staining was evaluated using FACS analysis. Data represents mean and standard deviation of three determinations. (**C**) WT and BAX/BAK DKO cells were stably transduced with a pool of lentiviral vectors expressing different shRNA constructs against *Perk* mRNA (shPERK) or control shRNA against the *luciferase* mRNA (shLuc). To determine the efficiency of knockdown, the level of phospho-PERK, and the downstream targets ATF4 and CHOP were assessed by Western blot in untreated cells (NT) or treated with 10 µg/ml Tm for 4 h. (**D**) Cells described in (C) were treated with Tm as described in (B) and cell death was monitored after 20 h by PI staining and FACS analysis. In addition, WT control cells were stably transduced with shPERK and shLuc lentiviral constructs for comparison in the same experiments. Data represents the mean and standard deviation of four independent experiments. *p* values were calculated with Student’s t-test (***: *p*<0.001, **: *p*<0.01 n.s.: non significant).

Based on the observation that mild serum withdrawal specifically restores the susceptibility of BAX/BAK DKO cells to ER stress-mediated apoptosis and not other intrinsic death stimuli (Figure 1G), we rationalized that signals emerging from the ER may regulate the engagement of mitochondrial-mediated apoptosis. To test this hypothesis we first analyzed the kinetics and intensity of early UPR signaling in WT and BAX/BAK DKO cells. Cells were exposed to Tm in the presence or absence of serum withdrawal, and the expression of major proximal UPR transcription factors, XBP-1 and ATF4, or the downstream transcription factor CHOP/GADD153 [24], [25], were followed over time by Western blot analysis. We did not observe any significant alteration of the kinetic of UPR signaling in DKO cells treated with 10 µg/ml Tm in the presence or absence of serum withdrawal (Figure 5A). This result is in agreement with our recent finding indicating that the regulation of XBP-1 expression by BAX and BAK or BH3-only proteins is lost when cells are treated with high Tm concentrations [38], [39]. More importantly, these results indicate that the intensity of ER stress is not altered by the serum withdrawal condition as monitored by the kinetics and amplitude of UPR signaling.

Activation of the UPR stress sensor IRE1α has been linked to ER stress-induced apoptosis through engagement of the JNK pathway [40], [41]. Treatment of cells with the selective JNK inhibitor SP600125 did not attenuate the rate of apoptosis of DKO cells exposed to Thap or Tm together with serum withdrawal (Figure 5B). Another death kinase, Glycogen Synthase Kinase-3 beta (GSK3-β), regulates ER stress-mediated apoptosis under certain conditions [42], [43]. Pre-treatments with two different GSK3-β inhibitors (TDZD-8 and L803-mts) did not affect the survival of BAX and BAK DKO cells undergoing ER stress in conjunction with mild serum withdrawal (Figure 5B).

Sustained activation of the ER stress sensor PERK triggers apoptosis [26], [44]–[47], whereas early signaling events promote cell protection [48], [49]. We knocked down PERK expression with shRNA and assessed the impact on cell death. An efficient down regulation of PERK was observed, reflecting an attenuation of PERK phosphorylation and the expression of ATF4 and CHOP/GADD153 (Figure 5C). Cell death induced by Tm treatment combined with serum withdrawal was significantly enhanced by targeting PERK expression (Figure 5D). This result suggest that UPR signaling modulates in part the occurrence of BAX and BAK-independent cell death after stimulation of ER stress under conditions of mild serum withdrawal.

### Cell Death in BAX/BAK DKO Cells is Independent of CypD Expression

**Figure 6:**
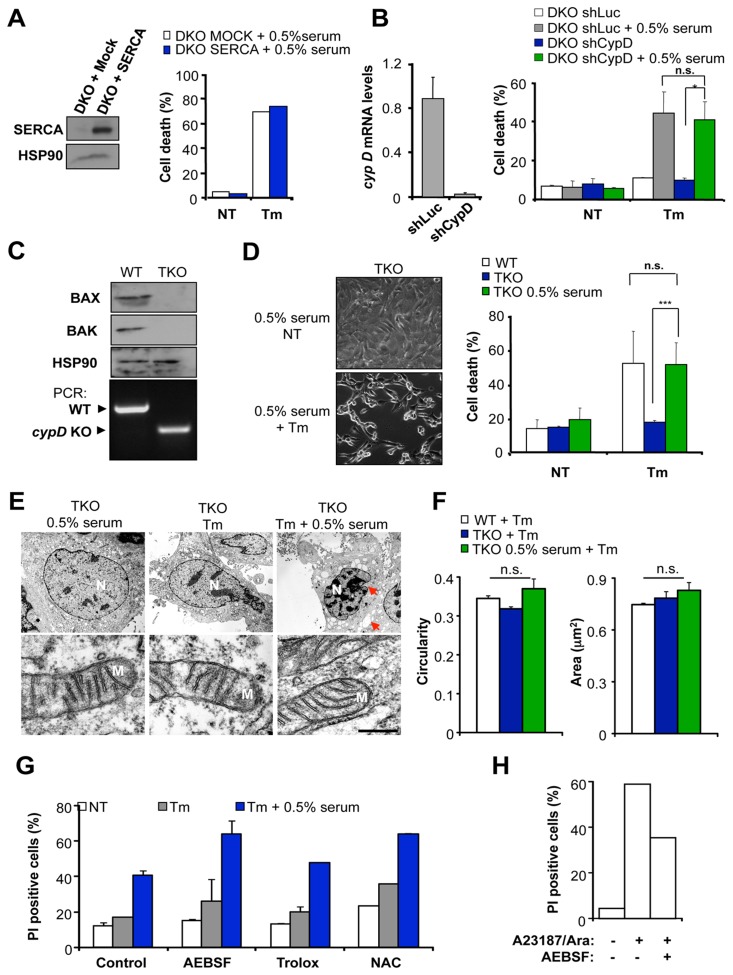
Cell death of BAX and BAK DKO cells is independent of CypD expression. (**A**) Right panel: BAX/BAK DKO cells stably transfected with a SERCA expression vector or an empty vector were treated with 10 µg/ml Tm or left untreated (NT) in cells pre-incubated for 2 h with cell culture media containing 0.5% serum. After 24 h, cell viability was analyzed by PI staining and FACS analysis. Left panel: SERCA expression levels were determined by Western blot analysis in the two cell lines presented. As loading control HSP90 levels were determined. Data is representative of two independent experiments. Mean is presented. (**B**) BAX/BAK DKO cells were stably transduced with lentiviral vectors expressing an shRNA against *cypD* mRNA (shCypD) or control shRNA against the *luciferase* mRNA (shLuc), and the mRNA levels of *cypD* were determined by real time PCR and normalized by the levels of *beta actin* (left panel). Then, in these cells, cell viability was analyzed after treatment with 10 µg/ml Tm in cells grown in regular cell culture conditions or pre-incubated for 2 h in media containing 0.5% serum. Untreated cells (NT) are also presented. After 24 h cell death was monitored by PI staining and FACS analysis (right panel). Mean and standard deviation is presented. *p* value for Tm treatment was calculated with Student’s t-test in a total of three independent experiments for indicated comparisons. n.s.: non significant (**C**) Confirmation of the genotype of BAX/BAK/CypD triple knockout (TKO) cells. Upper panel: The levels of BAX and BAK proteins were monitored by Western blot in TKO and WT control cells. HSP90 levels were used as loading control. Bottom panel: The *cypD* null genotype was assessed by PCR using genomic DNA of WT or TKO cells. PCR fragments corresponding to WT and *cypD* KO alleles are indicated. (**D**) Right panel: BAX/BAK/CypD TKO and parental control (WT) cells were analyzed as described in (**B**). Data represents mean and standard deviation of six independent experiments performed in duplicates. *p* value was calculated with Student’s t-test for indicated comparisons. n.s.: non significant. Left panel: phase contrast images to visualize morphological changes of TKO cells pre-incubated with cell culture media with 0.5% serum treated or not (NT) with 10 µg/ml Tm. (**E**) Upper panels: Cell morphology was monitored in BAX/BAK/CypD TKO cells treated as described in (D) using EM analysis. Arrowheads indicate classical apoptosis morphological features including chromatin condensation and fragmentations. In addition, cell shrinkage is observed in the analysis. N: nucleus. Lower panels: In the same cells presented in the upper panel, mitochondrial (M) morphology was visualized. A representative image is presented. (**F**) The area and circularity of mitochondria were determined in TKO cells in cells described in (**c**). Mean and standard deviation is presented of the analysis of 78 (0.5% serum), 105 (Tm) and 112 (Tm +0.5% serum) individual mitochondria per group respectively. (**G**) TKO cells were pre-treated with 100 µg/ml AEBSF, 1 mM Trolox or 5 mM N-acetylcysteine (NAC), for 30 min and then treated with 10 µg/ml Tm under regular growing conditions or in cells pre-treated for 2 h with media containing 0.5% serum. After 16 h, PI staining was evaluated using FACS analysis. (**H**) As positive control for the use of AEBSF, BAX and BAK DKO cells were treated with 100 µM arachidonic acid together with 10 µM A23187 in the presence or absence of 100 µg/ml AEBSF, and then after 16 h cell viability analyzed by PI staining and FACS analysis.

BAX/BAK DKO cells exhibit decreased steady state ER calcium content due to enhanced calcium leak, with concomitant resistance to PTP-induced cell death [27]. The ectopic expression of the ER-calcium pump ATPase SERCA in BAX/BAK DKO cells restores normal ER calcium levels, enhancing PTP-mediated apoptosis [27]. We did not observe any effects of SERCA overexpression in the occurrence of cell death after ER stress and serum withdrawal treatment in the same cells used in the previous study [27] (Figure 6A).

To test the role of CypD in the occurrence of BAX/BAK-independent apoptosis, we reduced the levels of *cypD* expression in BAX/BAK DKO cells with an efficient shRNA construct we recently described and validated [50] (Figure 6B, left panel). Stable knockdown of *cypD* mRNA in BAX/BAK DKO cells did not alter the occurrence of cell death after ER stress stimulation together with mild serum withdrawal (Figure 6B, right panel; and Figure S4A). Finally, to rule out the participation of CypD in this BAX/BAK-independent cell death pathway, we employed triple knockout (TKO) cells for *bax*, *bak*, and *cypD* (Figure 6C) previously described [22]. As expected, TKO cells were highly resistant to ER stress-induced cell death when compared with WT cells (Figure 6D and Figure S4B). Remarkably, this phenotype was completely reverted when ER stress stimulation was performed under conditions of mild serum withdrawal (Figure 6D and Figure S4B). In addition, the pharmacological inhibitor of cyclophilin D, cyclosporine A, was also used in our experimental system. Although DKO cells where not protected against ER stress/serum withdrawal, WT cells were partially protected against ER stress when pretreated with cyclosporine A (Figure S5). In addition, We also tested the impact of the VDAC inhibitor 4,49-diisothiocyanatostilbene-2,29-disulfonic acid (DIDS) [51], [52] on the death of TKO cells. 500 µM DIDS treatment did not block the induction of cell death of TKO cells by Tm and serum withdrawal co-treatments (not shown).

Cell death of TKO cells occurred through apoptosis as measured by evident cell shrinkage, chromatin condensation and fragmentation as observed by phase contrast (Figure 6D) or EM analysis (Figure 6E). Visualization of cells by EM did not reveal clear signs of mitochondrial swelling in TKO cells undergoing ER stress and serum withdrawal (Figure 6E). His observation was confirmed by measurements of mitochondrial area, perimeter or circularity (Fig. 6F and not shown). Since cytochrome c release and the engagement of intrinsic apoptosis of BAX/BAK DKO cells by arachidonic acid and A23187 co-treatments was reported to be fully blocked by the serine protease inhibitor 4-(2-Aminoethyl)-benzenesulfonyl fluoride hydrochloride (AEBSF) [22], we monitored the impact of AEBSF in our experimental system. Cell death induced by co-treatment of Tm with mild serum withdrawal was insensitive to 100 µg/ml AEBSF (Figure 6G). We confirmed the protection of AEBSF in the induction of cell death by arachidonic acid plus A23187 in BAX and BAK DKO cells (Figure 6H). Higher AEBSF concentrations were toxic to the cells (not shown). Finally, reactive oxygen species are well known mediators of cell death. Treatment of TKO cells with 1 mM Trolox or 5 mM N-acetylcysteine (NAC), two potent antioxidants, did not attenuate the death of TKO cells after Tm and serum withdrawal co-treatments (Figure 6G). These results suggest that BAX/BAK/CypD-independent cell death observed in our experimental system is not regulated by serine proteases.

## Discussion

In this article we have identified experimental conditions where classical intrinsic death stimuli engage the mitochondrial apoptosis machinery in the absence of BAX, BAK and CypD. BAX and BAK are fundamental components of the core apoptosis pathway upon which multiple death signals converge through activation/upregulation of specific BH3-only proteins to trigger cytochrome c release [2], [3], [53], [54]. *Bax* and *bak* double deficiency in mice is embryonic lethal due to failure of developmental programs that depend on apoptosis [4]. However, a small percentage of *bax* and *bak* DKO mice (∼10%) are viable [4], indicating that proper development can occur in the absence of these pro-apoptotic proteins. Whether developmental cell death in the absence of BAX and BAK is dependent on the CypD and mitochondrial PTP is unknown. We speculate that this may not be the case since most stimuli that trigger CypD-induced cell death rely on mitochondrial calcium uptake and production of reactive oxygen species, conditions more prompt to undergo necrotic cell death due to a rapid drop in ATP production. In fact, genetic ablation of *cypD* does not generate evident developmental defects, and does not alter the susceptibility to apoptosis of cells to a large number of known intrinsic death stimuli, but it modulated cell death after exposure to H_2_O_2_ (oxidative stress) *in vitro* and protected mice against brain and heart ischemia/reperfusion injury [16], [19], [55]. Our results, together with another report [22], suggest the existence of an alternative mitochondrial regulatory mechanism to instigate under certain circumstances the release of cytochrome c and apoptosome assembly in the absence of BAX/BAK and CypD. Similarly to Tsujimoto’s study [22], we can not exclude the possibility that Cyclosporin A-insensitive PTP (previously described *in vitro* [55]) operates in our experimental system to trigger cytochrome c release and apoptosis. However we did not detect any evidence for mitochondrial swelling in our EM analysis, a key feature of PRP-mediated cell death. A recent study described the activation of BAX/BAK-independent apoptosis when cell where deprived of glucose [56]. Apoptosis induction required RIPK1 and caspase-8 activation, but was independent of caspase-9 [57].

Irreversible ER stress leads to apoptosis, which requires the expression of BAX and/or BAK at the mitochondria [30]. Here we describe conditions in which mild serum withdrawal reestablishes the susceptibility of BAX/BAK DKO MEFs to undergo ER stress-dependent cell death in an apoptosome-dependent manner. Cell death was insensitive to BCL-X_L_ overexpression. Similar results were observed in cells double deficient for the upstream BH3-only proteins BIM and PUMA, where their resistance to ER stress was reverted by mild serum withdrawal. Unexpectedly, these effects were not observed when cell death was triggered with other intrinsic death stimuli such as DNA damage, inhibition of protein synthesis, or perturbations of microtubule dynamics, suggesting that a specific signal emerging from the ER may activate this alternative intrinsic death pathway.

**Figure 7:**
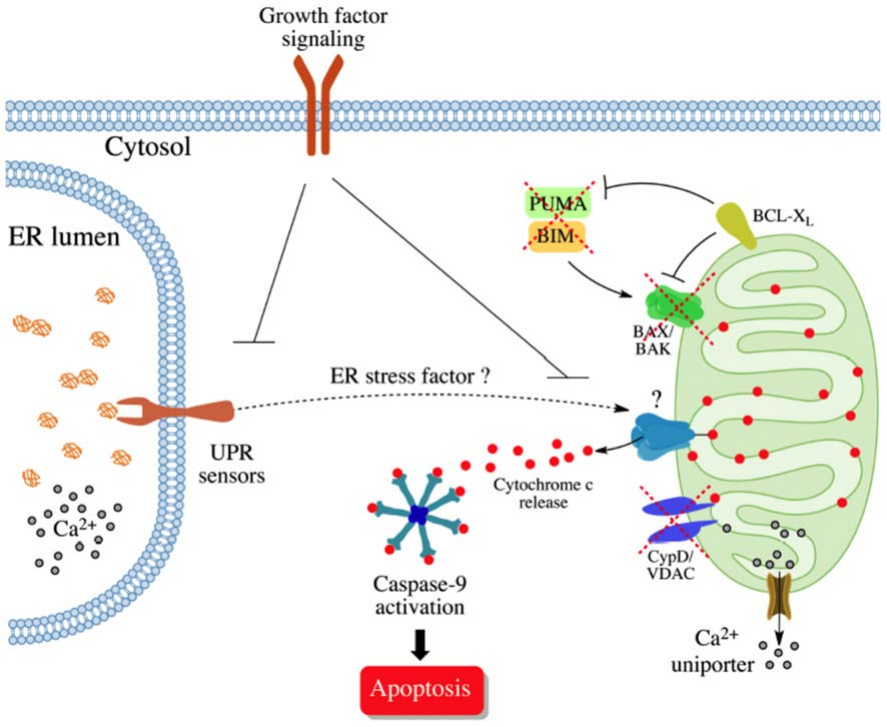
Working model: A BAX/BAK and CypD-independent intrinsic apoptosis pathway. ER stress-induced apoptosis is mediated by the activation of BAX and BAK at the mitochondria through upregulation of the upstream BH3-only proteins (i.e. BIM and PUMA) leading to mitochondrial outer membrane permeabilization, cytochrome c release, and subsequent apoptosome assembling. Under certain conditions such as mitochondrial calcium overload and oxidative stress, cytochrome c release can occur independently of BAX and BAK through opening of the mitochondrial permeability transition pore (PTP), which is form by several components including CypD and VDAC. Opening of the PTP leads to the expansion of the mitochondrial matrix, resulting in sufficient swelling to rupture the outer mitochondrial membrane and cytochrome *c* release. Stimulation of ER stress in conjunction with mild serum withdrawal triggers apoptosis in a BAX/BAK and CypD-independent manner. This alternative death pathway is mediated by the activation of caspase-9. Under normal conditions growth factor signaling may inhibit this alternative cell death pathway at the level of (i) mitochondria, or by (ii) blocking the activation of a specific ER-dependent event.

Since removal of serum from the cell culture media renders BAX/BAK/CypD TKO cells susceptible to ER stress-induced apoptosis, we speculate that trophic growth factor signaling may mask the activity of an alternative pathway to trigger apoptosis in a BAX/BAK/CypD-independent manner (model in Figure 7). Insulin/IGF1 signaling has been shown to inhibit cytochrome c release through activation of AKT, inducing the translocation of hexokinases to the mitochondrial membrane. Of note, this pathway controlled the release of cytochrome c even in the absence of BAX and BAK after exposition to UV light together with complete serum withdrawal. This phenomenon occurred only in a very small population of cells and with a slow kinetic of cytochrome c release [58], that is mediated by CypD [59]. We excluded the participation of this pathway in our experimental system since we were not able to protect BAX/BAK DKO cells against Tm/serum withdrawal-induced cell death after stimulating cells with insulin or IGF1 or treating with Phosphoinositide 3-kinases (PI3Ks) inhibitors (not shown). It remains to be determined how growth factor signaling blocks BAX/BAK/CypD-independent apoptosis. This inhibitory activity may operate at the level of the mitochondria to trigger cytochrome c release or it may block a yet unknown death-signaling event at the level of the ER (Figure 7). We are currently investigating additional models to uncover the molecular identity of this intrinsic BAX/BAK/CypD-independent death pathway. The possible involvement of lipids such as ceramides in the process constitute an interesting model since they form large lipid ion-channels *in vitro* [60] and can also induce the release of cytochrome c from purified mitochondria [61]. Moreover, ceramides and related lipids could mediate the crosstalk between ER and mitochondria in the induction of apoptosis in certain experimental systems [62]. The occurrence of BCL-2 family-independent and CypD-independent apoptosis may be taken into consideration when therapeutic strategies are designed to target this group of apoptosis regulators in a disease context, since depending on the cellular environment (i.e. presence of particular growth factors), and the cross-talk between different death signals, the way how apoptosis is regulated may drastically differ from our classical view of the process.

## Materials and Methods

### Materials

Tunicamycin (Tm), brefeldin A, thapsigargin, zVAD-fmk, zIETD-fmk, GSK3 inhibitors and Ruthenium red were purchased from Calbiochem EMB Bioscience Inc. IGF-1 was purchased from Australbiologicals and insulin from Actrapid. Cyclosporin A, etoposide, taxol, PI3K inhibitors, JNK inhibitors, TDZD-8 and L803-mts, TNF-α and PD98059 were obtained from Calbiochem. Cell medium, fetal calf serum and antibiotics were obtained from Life Technologies (Maryland, USA). Hoechst, 3,3-dihexyloxacarbocyanine [DIOC_6_(3)] and fluorescent secondary antibodies were purchased from Molecular Probes. Nocododazole, actinomycin D were obtained from Sigma. AEBSF was obtained from Roche.

### Cell Culture, Detection of Cell Death and Apoptosis

SV40 transformed MEFs were generated and cultured as described [29]. BAX and BAK DKO cells expressing SERCA or mitochondrial targeted BAX were previously described [27]. BAX/BAK/cyclophilin D triple knockout MEFs were previously described [22] and were kindly provided by Dr. Tsujimoto. Apoptosis was determined by FACS analysis of cells stained with Annexin-V FITC and Propidium Iodide (Promega) as previously described [29]. Analysis was performed using the Cell Quest program. Hypodiploid cells were quantified by FACS as previously described [63]. In parallel, nuclear morphology was analyzed by Hoechst33342 staining (Molecular Probes). To address the impact of serum withdrawal on ER stress-induced apoptosis, before treatment with ER stress agents, cells were pre-incubated with cell culture media containing 0.5% serum (fetal calf serum) for 2 h and then treated with brefeldin A, tunicamycin or thapsigargin. For caspase inhibition, cells were pre-treated with 10 µM zVAD-fmk for 30 min. Alternatively, cell viability was quantified in 96-well plates using 3-(4,5-dimethylthazol-2-yl)-5-3-carboxymethoxy-phenyl)-2-(4-sulfophenyl)-2H-tetrazolium (MTS) and phenazine methosulfate according to the recommendations of the supplier (CellTiter 96 AQueous; Promega, Madison, WI). Mitochondria targeted HA-BAX (mtBAX) was constructed by fusing the 29 amino acid mitochondrial targeted sequence from subunit VIII of human cytochrome *c* oxidase (COX VIII) into the N-terminal end of HA-BAX and was present on the OMM surface [27]. Retroviral expression vectors for HA-tagged BH3-only proteins were previously described [7]. The production of amphitropic retroviruses using the 293GPG packing cell line was performed as described previously [29]. Retroviral plasmids were transfected using Effectene (Qiagene) according to the manufacturer’s protocols. Retroviral transduction of each indicated BCL-2 family protein was confirmed by Western blotting using an anti-HA antibody.

### Knockdown of CypD, Caspases-9 and PERK

We generated stable MEFs with reduced levels of *cypD*, *caspases-9* and *perk* mRNA using methods previously described [38] by targeting each mRNA with shRNA using the lentiviral expression vector pLKO.1 and puromycin selection. As control a shRNA against the *luciferase* gene was employed. Constructs were generated by The Broad Institute (Boston, USA) based on different criteria for shRNA design (see http://www.broad.mit.edu/genome_bio/trc/rnai.html). For each gene we screened a total of five different constructs and selected the most efficient using real time PCR analysis. We identified the following targeting sequences for *caspase-9* (CCTTTGTTCATCTCCTGCTTA) and *cypD* (GCAGAATTGCTTAAAGTCAAA). For knocking down PERK a mix of five constructs was employed since none of the five targeting sequences alone were efficient to down regulate PERK. Targeting sequences are CCTCTACTGTTCACTCAGAAA, CCATGAGTTCATCTGGAACAA, GCCTGTTTGATGATACAAGTT, CCATACGATAACGGTTACTAT and GCCACTTTGAACTTCGGTATA.

### SDS-PAGE and Western Blot Analysis

Cells were homogenized by sonication on ice in RIPA buffer (20 mM Tris pH 8.0, 150 mM NaCl, 0.1% SDS, 0.5% DOC, 0.5% triton X-100) containing a protease inhibitor cocktail (Roche, Basel, Germany), NaF 1 mM, sodium orthovanadate and a phosphatase inhibitor cocktail (Promega). Protein concentration was determined by micro-BCA assay (Pierce, Rockford, IL). The equivalent of 30–50 µg of total protein was generally loaded onto 10% or 4–12% SDS-PAGE minigels (Cambrex) and analyzed by Western blotting. The following antibodies and dilutions were used: anti-CHOP/GADD153 1:2000, anti-ATF4 1:2000; anti-XBP-1 1:2000; anti-JNK1 1:5000 (Santa Cruz); anti-caspase-12 1:3000 (Exalpha Biologicals), anti-BAX and anti-BAK 1∶1000 (Upstate Technology); anti-HA 1∶2000 (Roche), anti-caspase-9 1:1000 (Pharmigen), anti-actin 1∶5000 (Chemicon Inc.), anti-SERCA 1∶1000 (Affinity Bioreagents), anti-HSP90 1:1000 (Santa Cruz Biotechnology) and anti-pPERK, and p-JNK 1∶2000 (Cell Signaling). After incubation with the primary antibody overnight at 4°C, membranes were washed and incubated for 1 h at room temperature with horseradish peroxidase-coupled secondary antibodies (Amersham Biosciences) diluted 1∶5000 in washing buffer. After washing, specifically bound antibodies were detected by enhanced chemiluminescence assay (Amersham Biosciences).

### Cytochrome c Release, Caspase Activation and Mitochondrial Membrane Potential Determination

WT, and DKO cells were washed 3 times with cold PBS and then fixed for 30 min with 4% formaldehyde on ice. Immunofluorescence to detect cytochrome c was performed with methods previously described [63] using an anti-cytocrome c antibody (Pharmingen) and fluorescent staining. Then cells were maintained in PBS containing 0.4% formaldehyde for visualization on a confocal microscope and analysis with the IP lab v 4.04 software (Beckon and Dickenson). In cells treated with ER stressors the mitochondrial membrane potential and cellular volume was analyzed by flow cytometry (FACS; Becton Dickinson, Mountain View, CA) after staining of the cells with 2 nM 3,3-dihexyloxacarbocyanine [DiOC_6_(3)] (Molecular Probes) [63]. For cleaved caspase-3 and cleaved caspase-9 immunofluorescence, anti-body were used 1∶250 (#95095 and #96615, Cell signaling). For mitochondrial and cytochrome C (1∶250, BD Pharmigen) colocalization determinations one focal plane was analyzed. The images obtained were deconvolved and the background was subtracted. Colocalization between organelles was quantified using the Mandeŕs algorithm, as previously described [64]–[66].

### Electron Microscopy Analysis

Cells were fixed in 2.5% glutaraldehyde, 0.01% picric acid, 0.1 M cacodylate buffer (pH 7.4) for 1 h, rinsed in the same buffer and incubated in 1% OsO_4_ for 30 minutes followed by incubation with 2% uranyl acetate. Cells were dehydrated with a graded series of ethanol, and infiltrated with Epon (Ted Pella Inc., #18005). Ultrathin sections were contrasted with 1% uranyl acetate and lead citrate. Grids were examined with a Philips Tecnai 12 electron microscope operated at 80 kV. Negative films were developed and scanned.

For quantification of mitochondrial morphology, photoshop software was used to quantify mitochondrial area, perimeter, circularity and mean gray value in differents treatments as we recently described [50].

### RNA Extraction and RT-PCR

Total RNA was prepared from cells using trizol (Invitrogen, Carlsbad, CA) and cDNA was synthesized with SuperScript III (Invitrogen, Carlsbad, CA) using random primers p(dN)_6_ (Roche, Basel, Switzerland). Quantitative real-time PCR reactions employing SYBR green fluorescent reagent were performed in an ABI PRISM 7700 system (Applied Biosystems, Foster City, CA). The relative amounts of mRNAs were calculated from the values of comparative threshold cycle by using β-actin as control. Primer sequences were designed by Primer Express software (Applied Biosystems, Foster City, CA) or obtained from the Primer Data bank (http://pga.mgh.harvard.edu/primerbank/index.html). Real time PCR was performed as previously described [38] using the following primers: *cyclophilin D* 5′-GGGACAGGTGGCGAAAGTATT-3′ and 5′-TTCGTATTGGGGCCTGCATTT-3′; *bim* 5′-CGACAGTCTCAGGAGGAACC-3′ and 5′-CATTTGCAAACACCCTCCTT-3′; *puma* 5′-GCCCAGCAGCACTTAGAGTC-3′ and 5′-GGTGTCGATGCTGCTCTTCT-3′; *noxa* 5′- GGAGTGCACCGGACATAACT-3′ and 5′-TTGAGCACACTCGTCCTTCA-3′; *caspases-9*, 5′-TCCTGGTACATCGAGACCTTG-3′and 5′-AAGTCCCTTTCGCAGAAACAG-3′, *actin* 5′-TACCACCATGTACCCAGGCA-3′ and 5-‘CTCAGGAGGAGCAATGATCTTGAT-3′.

## Supporting Information

Figure S1 **Serum withdrawal recovers the susceptibility of BAX and BAK DKO cells to ER stress-induced cell death.** WT MEFs were treated with 10 mg/ml Tm, 10 mM Thap or 20 mM brefeldin A in cells grown in regular cell culture media or in media containing 0.5% serum. Mild serum withdrawal was performed 2 h before the addition of ER stress agents. After 24 h of treatment, cell viability was analyzed with the MTS assay. Results are representative of two independent experiments performed in triplicate. Mean and standard deviations are presented of a representative experiment.
(TIF)

Figure S2 **BCL-XL in the control of cytocrome c redistribution and BH3-only protein expression and efficiency of retroviral transduction.** BAX/BAK DKO MEFs that stably transduced with retroviruses expressing human BCL-X_L_ or empty vector (Mock) cells were exposed to 20 µg/ml Tm in the presence of cell culture media containing 10% serum or pre-treated with media containing 0.5% serum. After 10 h, cells were stained with Mitotracker orange (red), fixed and cytochrome C (Cyt C, green) distribution visualized by indirect immuofluorescence. Images were acquired by confocal microscopy. Then, quantification of the Mandeŕs colocalization coefficient MTO/CytC (fraction of mitrotracker orange signal in co-localizing with cytochrome C signal) or CytC/MTO (fraction of the cytochrome C co-localizing mitotracker orange) were analyzed. Data represent the average and standard deviation of four independent experiments. One-way ANOVA test was used to analyze statistical significance (*: *p*<0.05; **: *p*<0.01; ***: *p*<0.01). (**B**) The expression of the HA-tagged BH3-only proteins was analyzed by Western blot after transfection of 293T packaging cells with pMIG-GFP retroviral vectors together with packaging vector to produce retroviruses employed in Figure 4F. Red arrowheads indicate the protein bands corresponding to each BH3-only protein. A HA-BAX expression vector was also used as control. (**B**) The percentage of retroviral transduction efficiency was assessed in WT and BAX/BAK DKO cells by monitoring the bicistronic expression of GFP by FACS analysis. Results are representative of two independent experiments performed in duplicated. Means are presented of a representative experiment.
(TIF)

Figure S3 **Low mRNA levels of pro-apoptotic protein BOK in WT and BAX/BAK DKO cells.** WT and BAX/BAK DKO cells were treated with 10 mg/ml Tm or left untreated. DKO cells were also pre-incubated for 2 h in cell culture media containing 0.5% serum. After 6 h of treatment, *bok* mRNA levels were assessed by real time PCR. As control, the levels of *bok* mRNA expression were also monitored in mRNA extracts from mouse ovary.
(TIF)

Figure S4 **Cell death in BAX and BAK DKO cells is independent of CypD.** (**A**) BAX/BAK DKO cells were stably transduced with lentiviral vectors expressing an shRNA against *cypD* mRNA (shCypD) or control shRNA against the *luciferase* mRNA (shLuc). Then, cell viability was analyzed after treatment with 10 mM Thap in cells grown in regular cell culture conditions or pre-incubated for 2 h in media containing 0.5% serum. Untreated cells (NT) are also presented. After 24 h cell death was monitored by PI staining and FACS analysis. (**B**) WT and BAX/BAK/CypD TKO cells were treated with 10 mM Thap under regular cell growing conditions or in cells pre-treated with media containing 0.5% serum for 2 h. After 24 h, cell death was analyzed by PI staining and FACS analysis. Mean and standard deviation are presented of three determinations.
(TIF)

Figure S5 **Effect of Cyclosporine A in cell death in WT and BAX and BAK DKO cells exposed to ER stress.** (**A**) BAX/BAK WT cells were or not pre-treated with CsA 10 mM for 30 min. Then, cells were incubated with 0.25, 1 and 2,5 mg/mL Tm for 24 h. Cell viability was analyzed by PI staining and FACS analysis. Data is representative of three independent experiments. Mean and standard deviation are presented. Significant differences were obtained between indicated control and experimental groups using Student’s t-test (** *p*<0.01). (**B**) BAX/BAK DKO cells were pre-treated with cell culture media containing 0.5% serum for 2 h in the presence or absence of 1 mM of CsA. Then, cells were exposed to Tm or Thap for 24 h. Cell viability was analyzed by PI staining and FACS analysis. Data is representative of two independent experiments. Mean is presented.
(TIF)
