# Supplementary figures and images for: A BAX/BAK and Cyclophilin D-Independent Intrinsic Apoptosis Pathway
Source: PLoS One. 2012 Jun 12;7(6):e37782. doi: 10.1371/journal.pone.0037782 (PMC3373601; doi:10.1371/journal.pone.0037782)

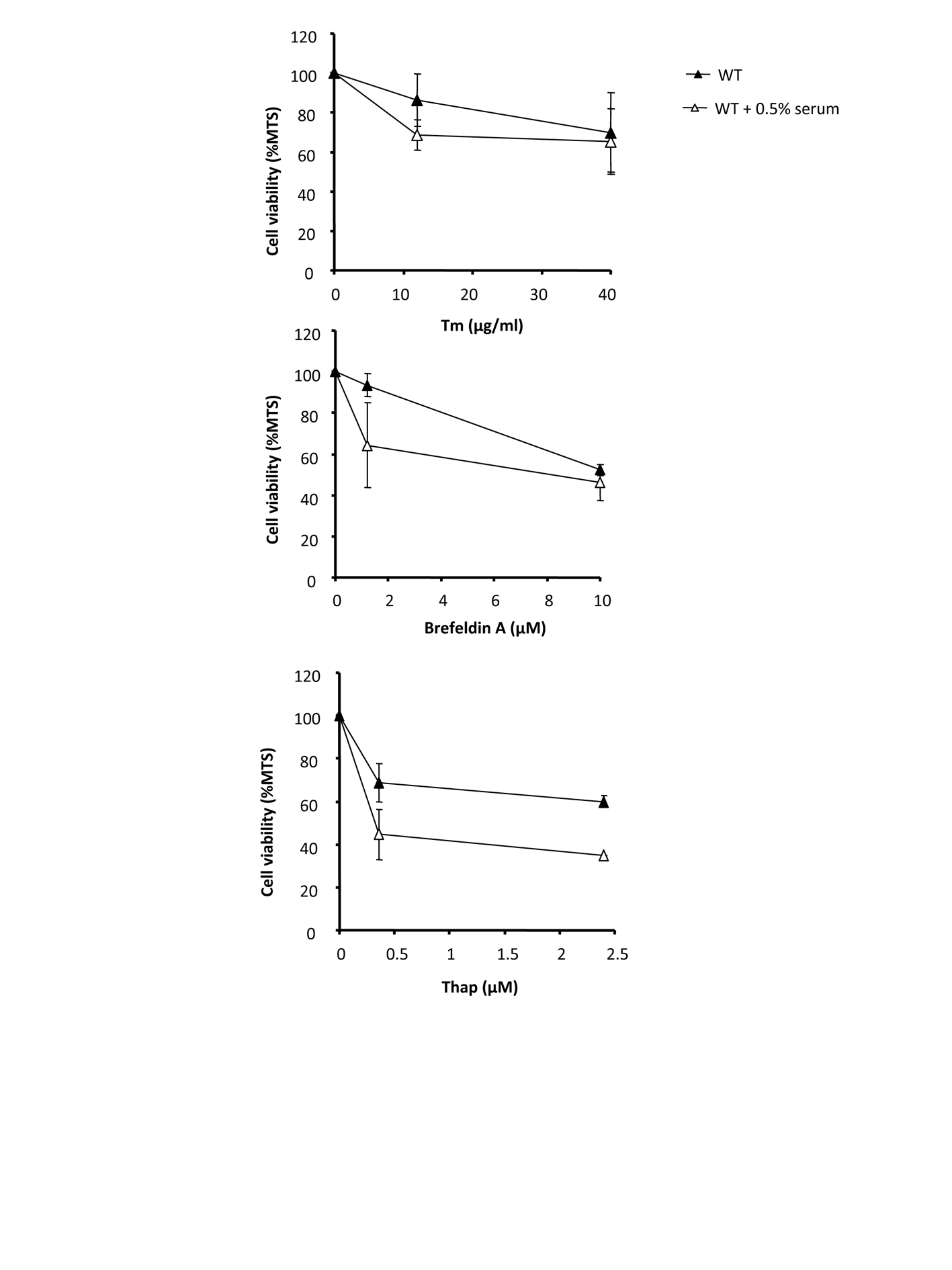

Supplement: Figure S1 — Serum withdrawal recovers the susceptibility of BAX and BAK DKO cells to ER stress-induced cell death. WT MEFs were treated with 10 mg/ml Tm, 10 mM Thap or 20 mM brefeldin A in cells grown in regular cell culture media or in media containing 0.5% serum. Mild serum withdrawal was performed 2 h before the addition of ER stress agents. After 24 h of treatment, cell viability was analyzed with the MTS assay. Results are representative of two independent experiments performed in triplicate. Mean and standard deviations are presented of a representative experiment. (TIF) [file pone.0037782.s001.tif]

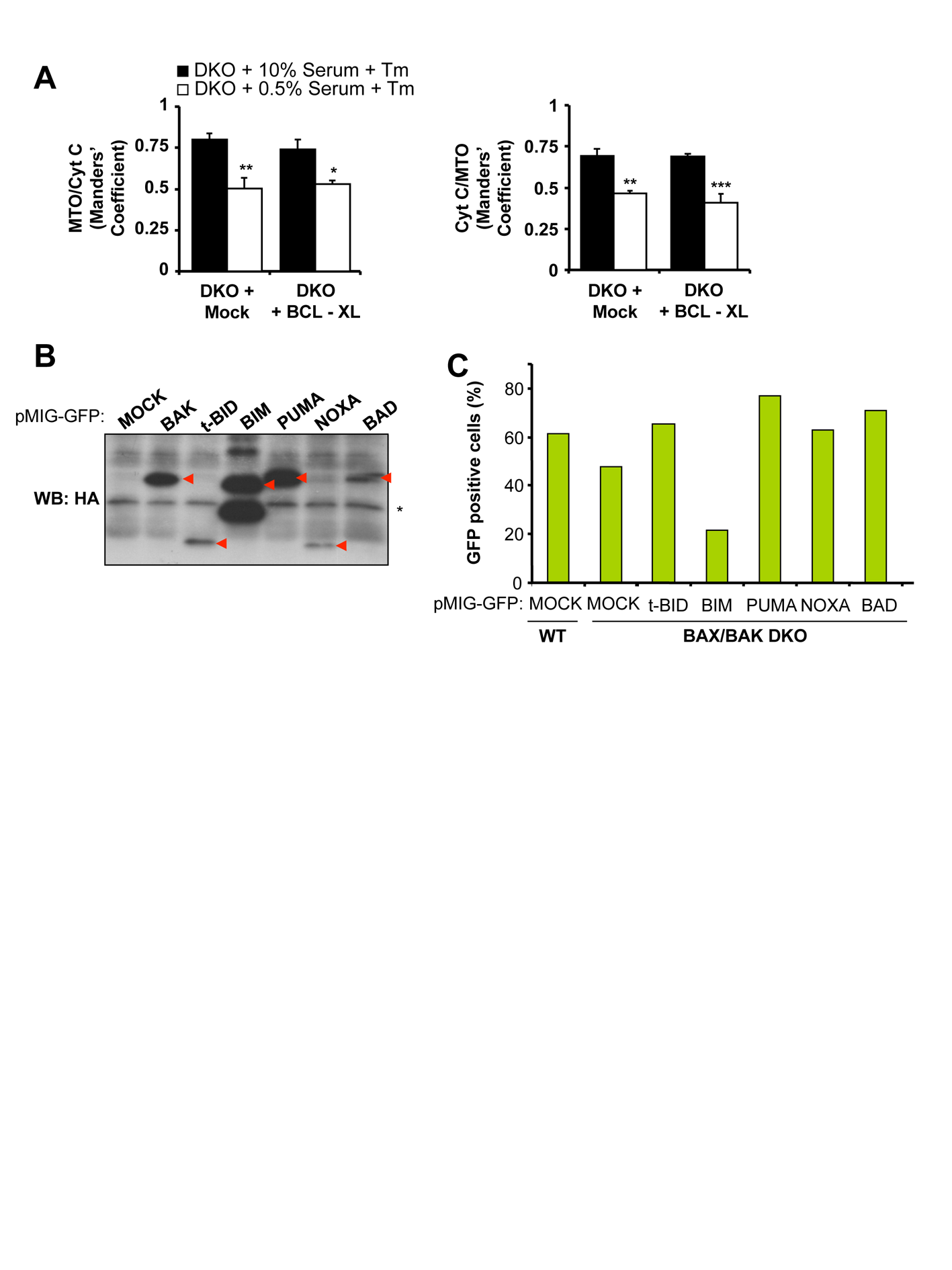

Supplement: Figure S2 — BCL-XL in the control of cytocrome c redistribution and BH3-only protein expression and efficiency of retroviral transduction. BAX/BAK DKO MEFs that stably transduced with retroviruses expressing human BCL-XL or empty vector (Mock) cells were exposed to 20 µg/ml Tm in the presence of cell culture media containing 10% serum or pre-treated with media containing 0.5% serum. After 10 h, cells were stained with Mitotracker orange (red), fixed and cytochrome C (Cyt C, green) distribution visualized by indirect immuofluorescence. Images were acquired by confocal microscopy. Then, quantification of the Mandeŕs colocalization coefficient MTO/CytC (fraction of mitrotracker orange signal in co-localizing with cytochrome C signal) or CytC/MTO (fraction of the cytochrome C co-localizing mitotracker orange) were analyzed. Data represent the average and standard deviation of four independent experiments. One-way ANOVA test was used to analyze statistical significance (*: p<0.05; **: p<0.01; ***: p<0.01). (B) The expression of the HA-tagged BH3-only proteins was analyzed by Western blot after transfection of 293T packaging cells with pMIG-GFP retroviral vectors together with packaging vector to produce retroviruses employed in Figure 4F. Red arrowheads indicate the protein bands corresponding to each BH3-only protein. A HA-BAX expression vector was also used as control. (B) The percentage of retroviral transduction efficiency was assessed in WT and BAX/BAK DKO cells by monitoring the bicistronic expression of GFP by FACS analysis. Results are representative of two independent experiments performed in duplicated. Means are presented of a representative experiment. (TIF) [file pone.0037782.s002.tif]

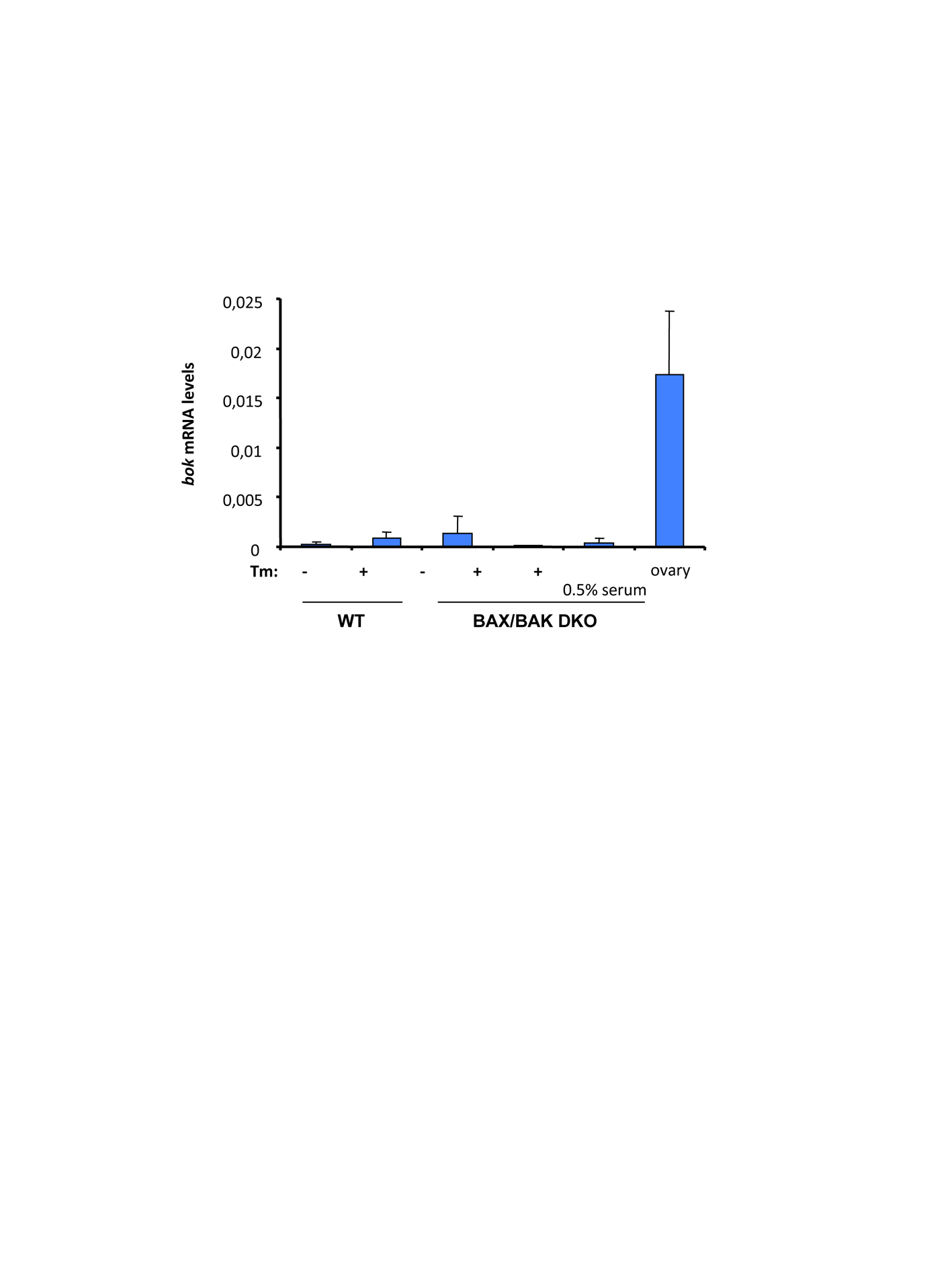

Supplement: Figure S3 — Low mRNA levels of pro-apoptotic protein BOK in WT and BAX/BAK DKO cells. WT and BAX/BAK DKO cells were treated with 10 mg/ml Tm or left untreated. DKO cells were also pre-incubated for 2 h in cell culture media containing 0.5% serum. After 6 h of treatment, bok mRNA levels were assessed by real time PCR. As control, the levels of bok mRNA expression were also monitored in mRNA extracts from mouse ovary. (TIF) [file pone.0037782.s003.tif]

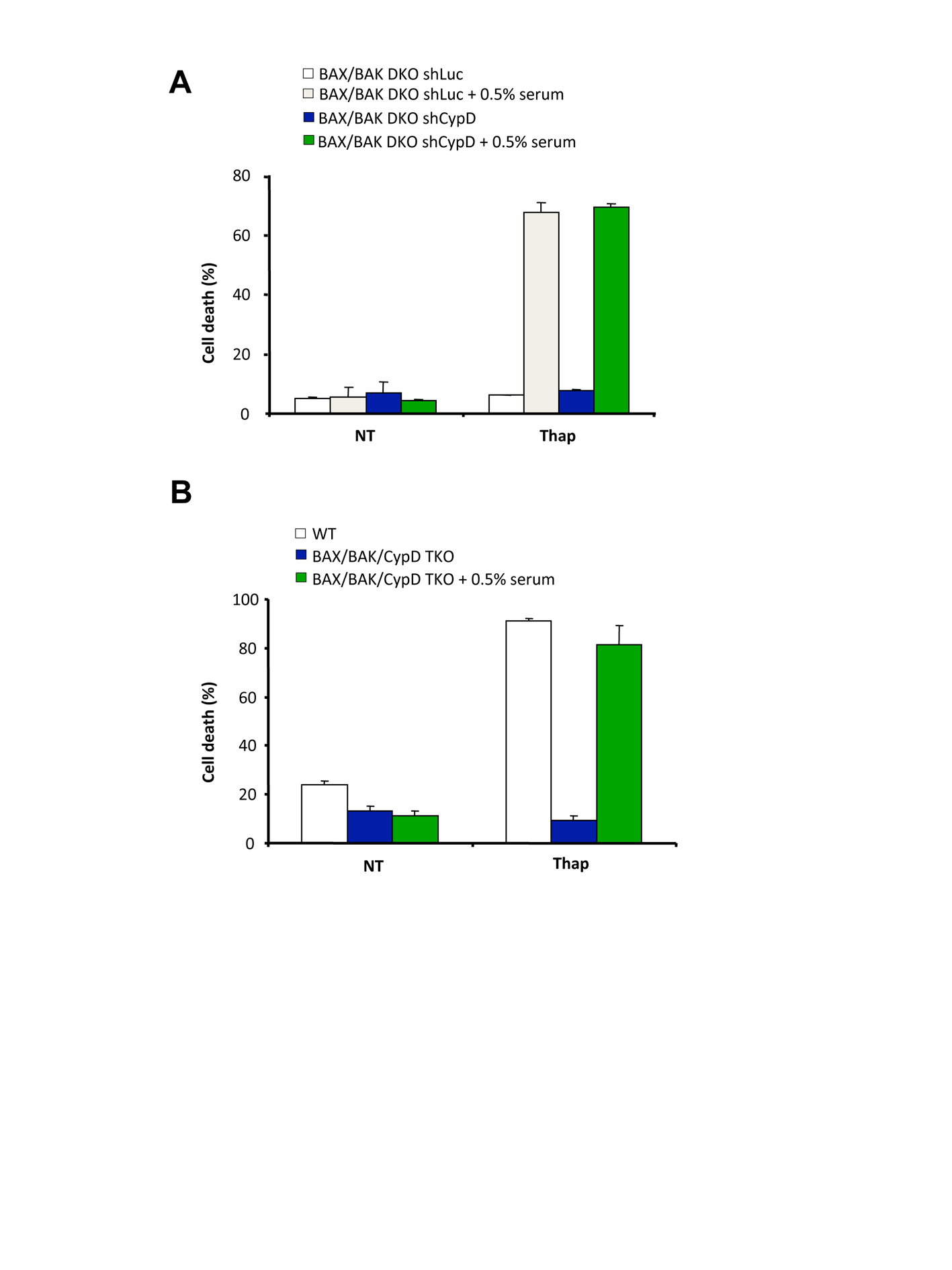

Supplement: Figure S4 — Cell death in BAX and BAK DKO cells is independent of CypD. (A) BAX/BAK DKO cells were stably transduced with lentiviral vectors expressing an shRNA against cypD mRNA (shCypD) or control shRNA against the luciferase mRNA (shLuc). Then, cell viability was analyzed after treatment with 10 mM Thap in cells grown in regular cell culture conditions or pre-incubated for 2 h in media containing 0.5% serum. Untreated cells (NT) are also presented. After 24 h cell death was monitored by PI staining and FACS analysis. (B) WT and BAX/BAK/CypD TKO cells were treated with 10 mM Thap under regular cell growing conditions or in cells pre-treated with media containing 0.5% serum for 2 h. After 24 h, cell death was analyzed by PI staining and FACS analysis. Mean and standard deviation are presented of three determinations. (TIF) [file pone.0037782.s004.tif]

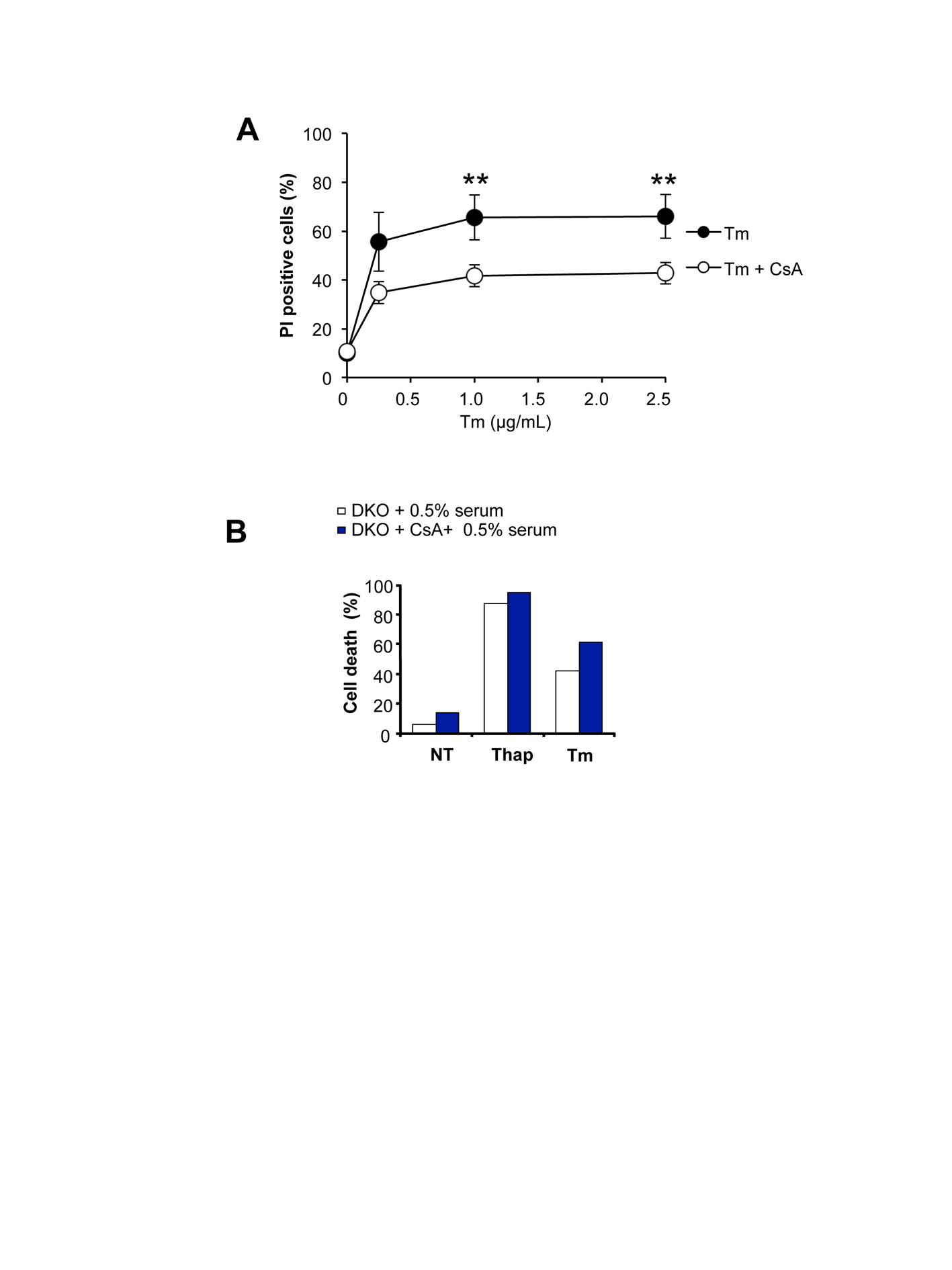

Supplement: Figure S5 — Effect of Cyclosporine A in cell death in WT and BAX and BAK DKO cells exposed to ER stress. (A) BAX/BAK WT cells were or not pre-treated with CsA 10 mM for 30 min. Then, cells were incubated with 0.25, 1 and 2,5 mg/mL Tm for 24 h. Cell viability was analyzed by PI staining and FACS analysis. Data is representative of three independent experiments. Mean and standard deviation are presented. Significant differences were obtained between indicated control and experimental groups using Student’s t-test (** p<0.01). (B) BAX/BAK DKO cells were pre-treated with cell culture media containing 0.5% serum for 2 h in the presence or absence of 1 mM of CsA. Then, cells were exposed to Tm or Thap for 24 h. Cell viability was analyzed by PI staining and FACS analysis. Data is representative of two independent experiments. Mean is presented. (TIF) [file pone.0037782.s005.tif]
